# Visualization and analysis of PM_2.5_ health effects, 2013 to 2023: Bibliometrics of PM_2.5_ and health effects

**DOI:** 10.1097/MD.0000000000040793

**Published:** 2024-12-06

**Authors:** Feifei Huang, Lin Zhou, Yao Lu, Ruiwei Liao, Yan Li, Ming Li

**Affiliations:** a School of Basic Medical Sciences, Guangdong Pharmaceutical University, Guangdong, China, Guangzhou City, Guangdong Province, P.R. China; b School of Basic Medical Sciences, Guangzhou University of Chinese Medicine, Guangzhou City, Guangdong Province, P.R. China; c School of Life Science and Biopharmaceutics, Guangdong Pharmaceutical University, Guangzhou City, Guangdong Province, P.R. China.

**Keywords:** health effects, citespace, web of science, PM_2.5_

## Abstract

**Background:**

To analyze the current status, hotspots, and cutting-edge trends of PM_2.5_ health effects of PM_2.5_, using CiteSpace.

**Methods:**

The Web of Science Core Collection Science Citation Index Expanded was searched for relevant articles from January 1, 2013, to December 31, 2023. Network maps identifying authors, institutions, countries, keywords, co-cited authors, journals, references, and research trends were then created using CiteSpace.

**Results:**

A total of 2174 articles on the health effects of PM_2.5_ were identified between 2013 and 2023, with an increasing trend in annual publications. The United States had the highest number of articles on this topic, followed by China. The Chinese Academy of Sciences (CAS) is the leading institute in terms of paper production. Hoek was the most prolific author, focusing on the health consequences of air pollution. Among referenced journals, ENVIRON HEALTH PERSP ranked first, while Pope Ca was the most cited author. Current research focuses on reactive oxygen species (ROS), inflammation, oxidative stress, cardiovascular and respiratory diseases, PM_2.5_ sources, and chemical composition. The field is currently experiencing a phase of rapid expansion.

**Conclusion:**

The findings of this bibliometric analysis offer insight into the status and direction of research on PM_2.5_ and its health impacts, which aid scientists in coming up with new directions for their investigation.

## 1. Introduction

One important marker of air pollution introduced into the atmosphere by a range of human and natural activities is particulate matter (PM). Atmospheric PM is classified into 4 categories according to aerodynamic diameter: Total suspended particulate (TSP ≤ 100 μm), particulate matter (PM ≤ 10 μm), fine particulate matter (PM_2.5_ ≤ 2.5 μm), and ultrafine particles (UFPS ≤ 0.1 μm).^[1]^ The possibility of particles creating health issues has been strongly correlated with their size.^[2]^ PM_2.5_ refers to fine PM with a diameter less than or equal to 2.5 micrometers, which is so small that it can easily pass through the respiratory tract and enter the human body. Due to its tiny size, PM_2.5_ can penetrate deep into the tiny bronchial tubes and alveoli of the lungs, causing serious harm to human health.^[3]^ As per the Global Burden of Diseases Study 2015, long-term exposure to ambient PM_2.5_ was the fifth leading worldwide cause of mortality, responsible for 4.2 million deaths (95% uncertainty interval [UI] 3.7–4.8 million).^[4,5]^ Research has indicated that PM_2.5_ can cause respiratory diseases such as asthma and chronic obstructive pulmonary disease (COPD) when it enters the human body. Long-term exposure to high PM_2.5_ concentrations may also increase the risk of lung cancer.^[4,6,7]^ In addition, high concentrations of PM_2.5_ are positively correlated with cardiovascular disease morbidity and mortality, which may lead to impaired vascular endothelial function, cardiac arrhythmia, etc, increasing the risk of cardiovascular disease.^[8]^ Several studies have found that people who are chronically exposed to PM_2.5_ are more likely to experience cognitive decline and neurological disorders such as PD and AD disorders.^[9,10]^ Consequently, the correlation between PM_2.5_ and its impact on health is a hot subject of ongoing discussion, with a rapidly increasing number of published articles.

A bibliometric analysis is a useful tool for determining the general pattern of research activity and elucidating the relationships between pertinent research institutes.^[11]^ In addition, bibliometrics may assess the volume and trend of scientific output in important biomedical fields across nations and years, they are especially helpful for new fields whose effects on the broader field of biomedical research are still being completely assessed^.[12]^ So, the review methodology offers unique and unprecedented advantages that are difficult to find in traditional literature reviews. CiteSpace, as a bibliometric tool, has several advantages such as visual analysis, keyword analysis, collaboration network analysis, and so on. It can help researchers better understand the development trend of the academic research field and evaluate the effect of academic cooperation.^[13,14]^ Some researchers have used this method to summarize the research results on PM. For example, Jia et al^[15]^ summarized the mechanisms of damage to the cardiovascular system caused by PM. Wang et al^[16]^ organized and collected literature on the relationship between PM and atherosclerosis. Zhao et al^[17]^ used bibliometric methods to analyze the toxicity of indoor PM_2.5_.Although they analyzed PM_2.5_ and its impact on health, they used broader keywords, which may result in inaccurate results. Moreover, with the development of the economy and the improvement of living standards in recent years, people’s concern about PM_2.5_ and its effects on health has been increasing, which has led to a significant increase in the number of related research articles in the last decade. This research boom has also introduced new research perspectives and areas, highlighting the need for further literature reviews and metric studies in this area to provide researchers with up-to-date research references.

In this study, the Web of Science database was utilized to search for literature on the health effects of PM_2.5_ from 2013 to 2023. Citespace software was then employed to analyze and summarize the trends, initial findings, and research progress. The primary aim of our study was to guide researchers in exploring the health effects of PM_2.5_ through bibliometric analysis.

## 2. Methods

### 2.1. Ethical statement

This systematic review does not require ethical approval as it does not involve animal or human clinical trials and is not unethical.

### 2.2. Search strategy

We obtained papers from the Web of Science Core Collection Science Citation Index Expanded (SCI-Expanded) from January 1, 2013, to December 31, 2023, using the following terms: PM_2.5_, health effects. Table 1 provides a detailed search technique.

**Table 1:** Search strategy.

Search Query		Results
#1	TS = (PM2.5 OR “fine particulate matter” OR “fine particles”)	59,399
#2	TS = (“health effects” OR “human health effects”)	39,059
#3	(((#1 AND #2) AND DT = (Article)) AND LA = (English)) AND DOP = (2013-01-01/2023-12-31)	2194

### 2.3. Bibliometrics and visualization analysis

Create a CiteSpace folder with 4 subfolders: Input, Output, Data, and Projects. The articles retrieved from the Web of Science were exported in plain text format, full-text records and references were selected at the record content, and the file was named “ download_XXX.txt,” and then imported into CiteSpace 6.2. *R*6 for further analysis. After excluding duplicates, 2174 documents remained. We used CiteSpace’s primary procedural steps–time slicing, thresholding, modeling, pruning, merging, and mapping–when mapping and visualizing knowledge figures.^[13]^ In this paper, the time is set to “2013 to 2023,” selection criteria top N was set to 50, pruning selected pathfinder, pruning sliced networks simplified mapping. Three key ideas in CiteSpace are diverse networks, betweenness centrality, and burst detection. These ideas can be used to quickly depict hotspots, frontiers, and research status.^[14]^ Different maps’ nodes stand in for authors, institutions, nations, or keywords. Node size and color represent the frequency of occurrence and citation, respectively, as well as the years of occurrence and citation.^[18]^

## 3. Results

### 3.1. Annual publications analysis

**Figure 1.**
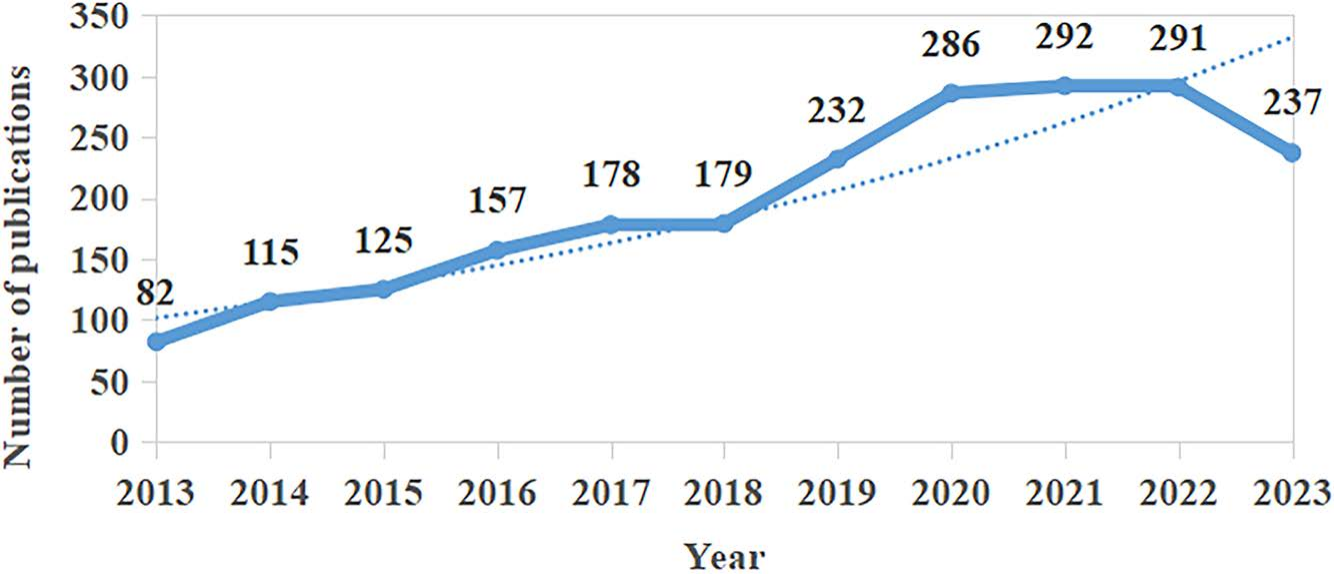
Annual trend of publications.

From 2013 to 2023, 2194 articles were retrieved, and 20 ineligible records were manually screened to exclude them, resulting in 2174 documents. As can be seen from Figure 1, the overall trend of the number of publications in the last decade has been upward, with the number of publications leveling off in 2013 to 2018, with no more than 50 per year, and the largest increase in the number of relevant publications in 2019 to 2020 (54 publications), peaking in 2021 (292 publications). Although there is a small decrease in 2022 to 2023, the number of articles is still over 200. All this indicates that more and more scholars are beginning to pay attention to this topic.

### 3.2. Countries and institutions analysis

**Figure 2.**
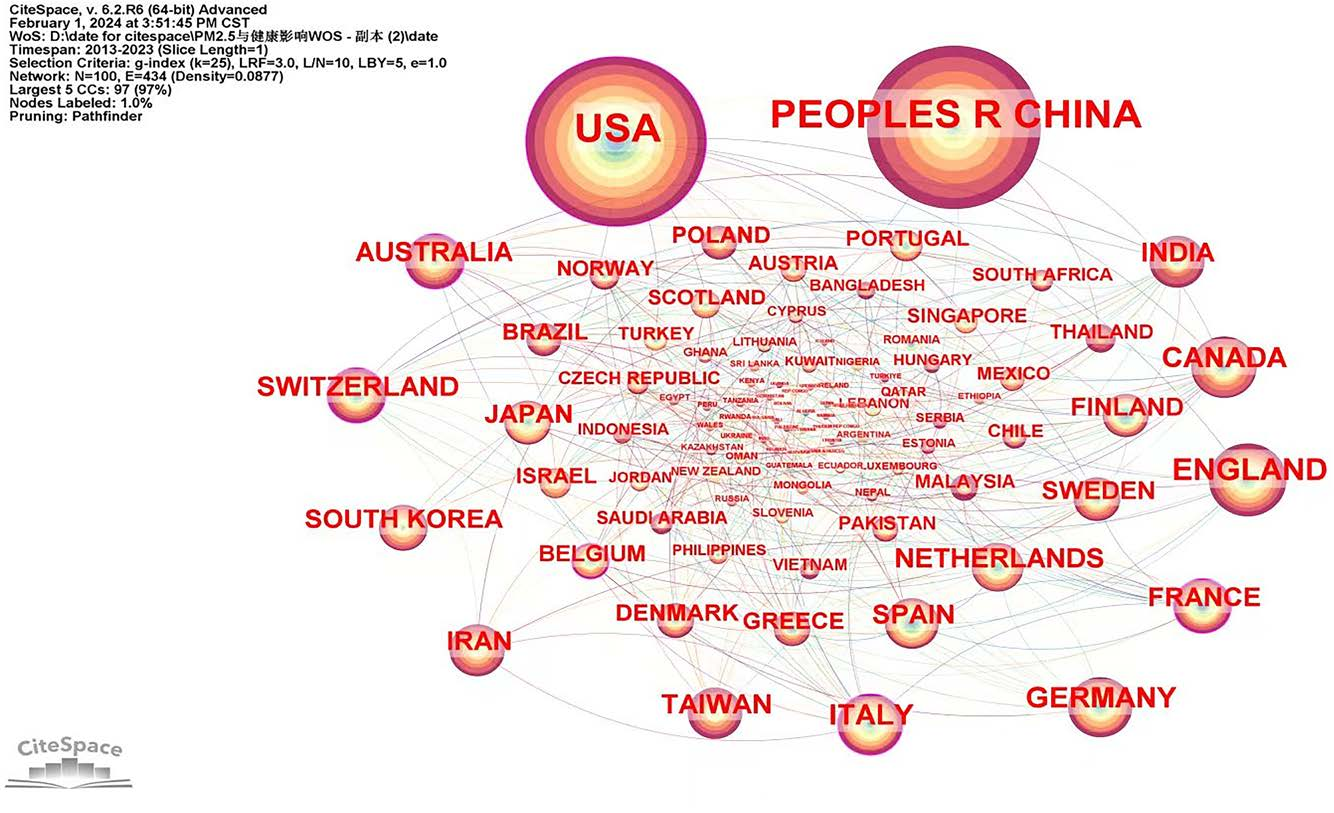
The network of countries.

**Figure 3.**
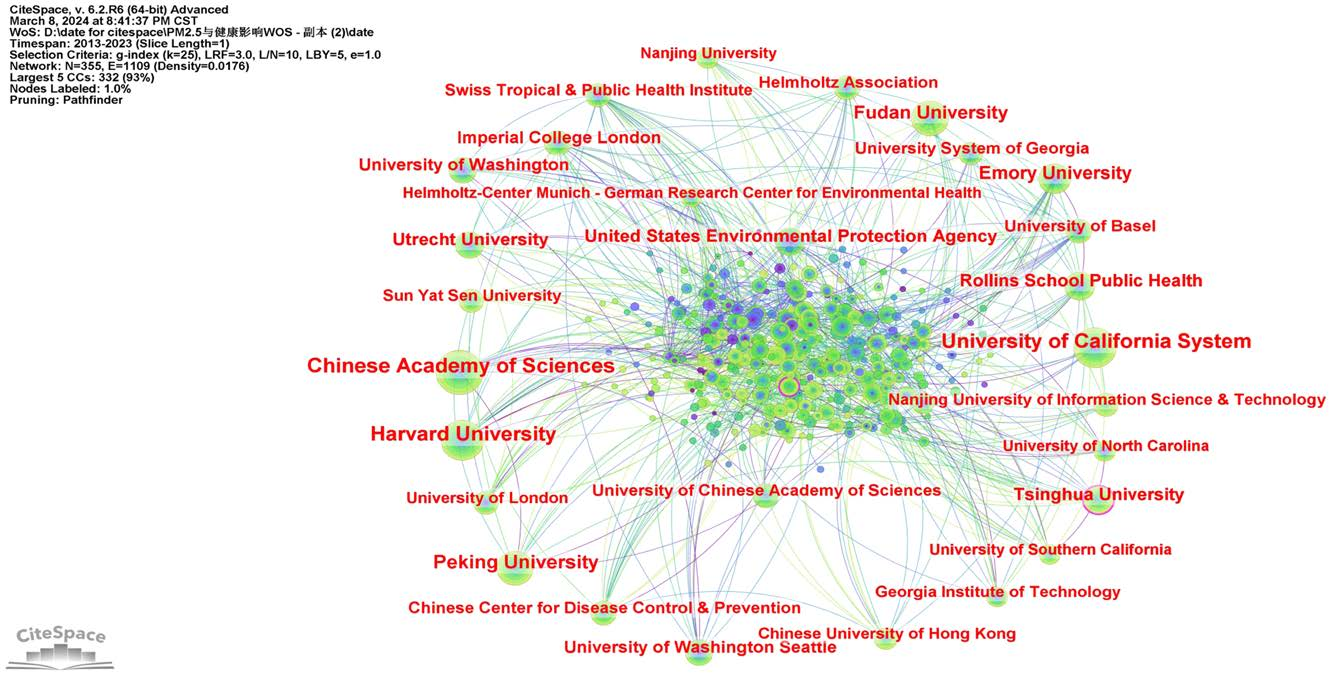
The network of institutions.

The cooperation network of countries and institutions is shown in Figures 2 and 3, respectively. The size of the nodes in the figures corresponds to the number of articles published by the countries or institutions, respectively. Larger nodes or name fonts indicate a higher number of published articles. Link lines between countries or institutions signify cooperation, with thicker lines denoting more frequent collaborations. The country’s cooperation analysis (Fig. 2 and Table 2) revealed node N = 100, link E = 434, and Density = 0.0877. The top 10 countries identified are the United States, China, England, Canada, Germany, Italy, Taiwan, Netherlands, Switzerland, and Australia. The top ten in terms of centrality are the United States, Italy, Switzerland, France, Belgium, Australia, Denmark, Canada, Germany, and Saudi Arabia. These findings suggest that these countries hold significant influence in research on the effects of PM_2.5_ on health.

**Table 2:** Top 10 countries in terms of citation frequency and centrality.

Rank	Country	N	Centrality	Rank	Country	N	Centrality
1	USA	807	0.17	1	USA	807	0.17
2	Peoples China	752	0.01	2	Italy	109	0.15
3	England	159	0.04	3	Switzerland	80	0.13
4	Canada	114	0.08	4	France	79	0.13
5	Germany	110	0.08	5	Belgium	36	0.13
6	Italy	109	0.15	6	Australia	80	0.12
7	Taiwan	87	0.02	7	Denmark	42	0.09
8	Netherlands	83	0.04	8	Canada	114	0.08
9	Switzerland	80	0.13	9	Germany	110	0.08
10	Australia	80	0.12	10	Saudi Arabia	15	0.08

The results of the institution cooperation analysis network, depicted in Figure 3, reveal that node N = 354, link E = 1111, and Density = 0.0178. As shown in Table 3, the top 10 institutions in the cooperative network are the CAS, University of California System, Harvard University, Peking University, Fudan University, Emory University, Utrecht University, United States Environmental Protection Agency, Tsinghua University, and Rollins School Public Health. The results also suggest that the CAS, the University of California System, and Harvard University hold significant importance and authority within the network. The United States has conducted extensive research on the health effects of PM_2.5_ to determine its adverse effects on health, such as increased risk of respiratory diseases, cardiovascular diseases, and cancer.

**Table 3:** The top ten cited institutions.

Rank	Frequency	Centrality	Year	Institution
1	134	0.09	2013	Chinese Academy of Sciences
2	116	0.02	2013	University of California System
3	114	0.09	2013	Harvard University
4	88	0.09	2013	Peking University
5	78	0.05	2013	Fudan University
6	70	0.05	2013	Emory University
7	62	0.03	2013	Utrecht University
8	60	0.03	2013	United States Environmental Protection Agency
9	57	0.1	2015	Tsinghua University
10	55	0.06	2013	Rollins School Public Health

### 3.3. coauthors and co-citation author network analysis

**Figure 4.**
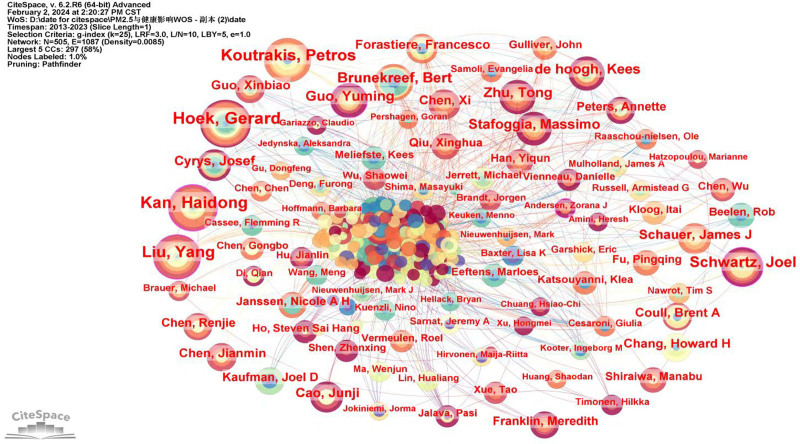
The network of coauthors.

In the analysis of author cooperation (results as shown in Fig. 4 and Table 4), the findings indicate a total of 505 authors and 1087 collaborations, with N = 505 nodes, link E = 1087, and a density of 0.0085. Notable authors include Hoek, Gerard (frequency = 34), Kan, Haidong (frequency = 31), Koutrakis, Petros (frequency = 29), Liu, Yang (frequency = 29), Schwartz, Joel (frequency = 29), and Brunekreef, Bert (frequency = 22). Notably, Professor Hoek from the University of Utrecht and Professor Kan from Fudan University have published the highest number of articles. Professor Hoek, Gerard assessed the effects of transportation policies on air pollution concentrations and respiratory health. The study found that the most significantly affected street was 1 with a significant reduction in traffic-related pollution, whose residents tended to have a slight improvement in respiratory function. The study suggests that air pollution reductions may have some positive impact on respiratory function, especially in areas with significant reductions in pollution.^[19]^ Professor Kan, Haidong’s Research interests are environmental science and ecology public, environmental and occupational health. Professor Kan, Haidong, and his team used stepwise regression to construct a season-specific model using climatic variables and questionnaire-based predictors and found that residential PM_2.5_ varied considerably between seasons, as well as between residences within seasons.^[20]^

**Table 4:** The top ten cited authors.

Rank	Frequency	Centrality	Year	Author
1	34	0.05	2014	Hoek, Gerard
2	31	0.21	2013	Kan, Haidong
3	29	0.04	2013	Koutrakis, Petros
4	29	0.16	2014	Liu, Yang
5	22	0.1	2013	Schwartz, Joel
6	19	0	2014	Brunekreef, Bert
7	17	0.14	2018	Guo, Yuming
8	17	0.01	2019	Zhu, Tong
9	17	0.01	2015	de Hoogh, Kees
10	16	0.06	2019	Stafoggia, Massimo

**Figure 5.**
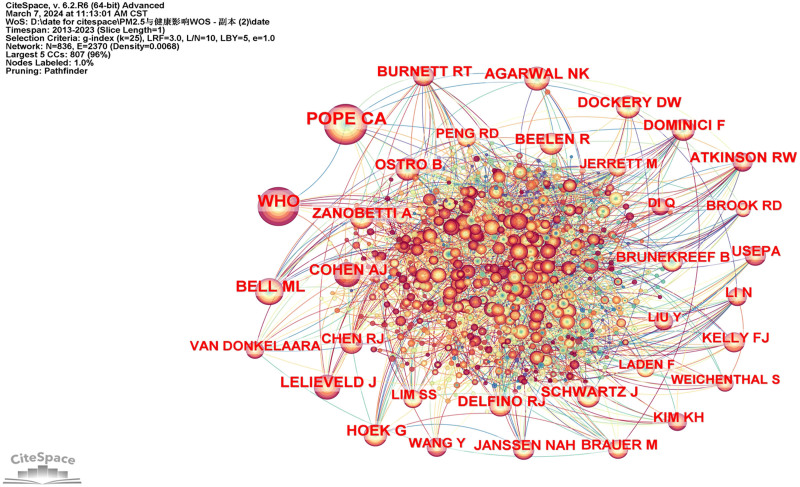
The network of co-cited authors.

Co-citation refers to the phenomenon that 2 or more authors or their articles are cited by other literature at the same time.^[21]^ Among the author co-citations, the most cited author was Pope Ca, with a citation frequency of 572, followed by WHO, and Bell Ml (Fig. 5 and Table 5).

**Table 5:** The top 10 co-cited author.

Rank	Co-citations	Centrality	Year	Co-cited authors
1	572	0.01	2013	Pope Ca
2	518	0.01	2013	WHO
3	252	0.03	2013	Bell ML
4	220	0.01	2013	Agarwal NK
5	212	0.01	2013	Cohen AJ
6	211	0.02	2013	Hoek G
7	202	0.01	2016	Lelieveld J
8	197	0.01	2013	Dockery DW
9	193	0.03	2013	Ostro B
10	189	0.04	2013	Zanobetti A

### 3.4. Co-citation analysis of journals and references

**Figure 6.**
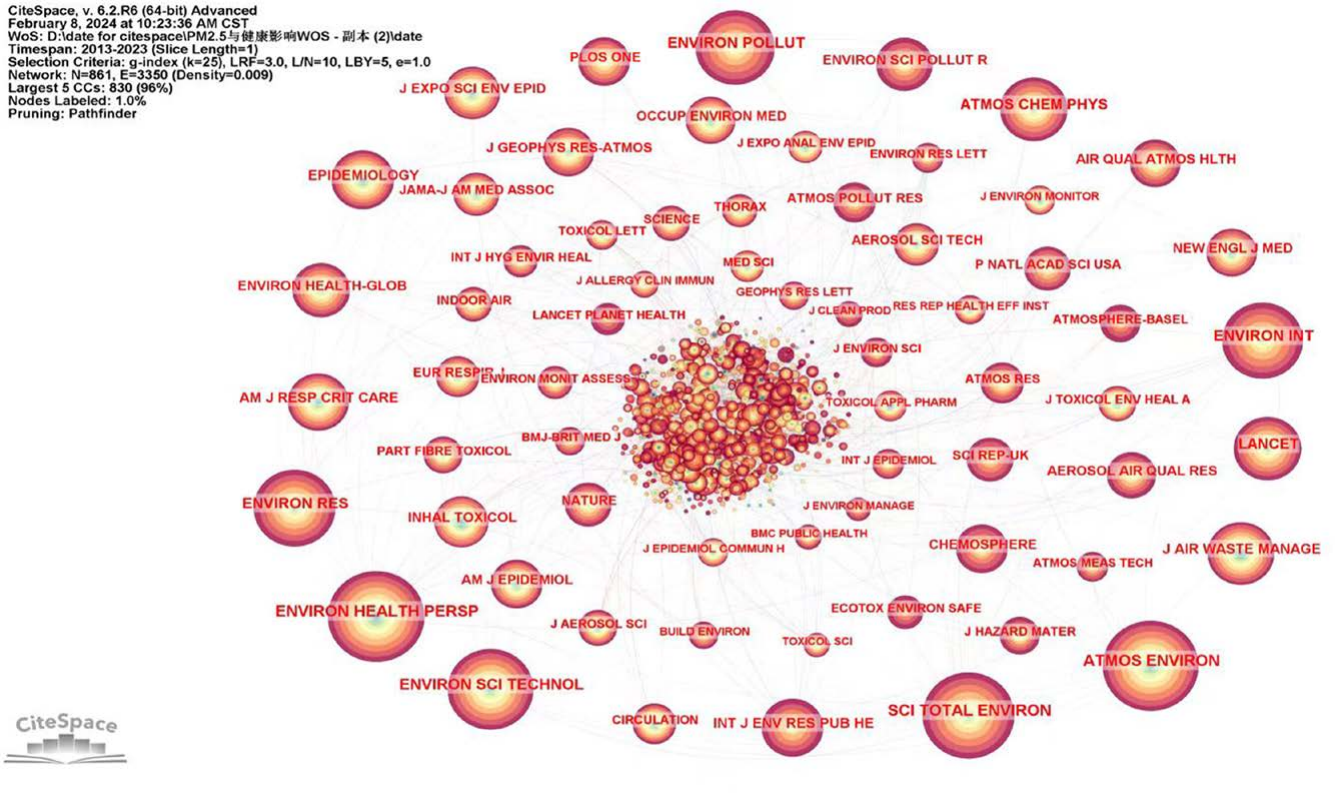
The network of co-cited journals.

Figure 6 shows the journal co-citation network map. Results showed that nodes N = 816, link E = 3350, and Density = 0.009. The node size is a representation of each journal’s co-citation frequency. Table 6 lists the top 10 most frequently co-cited journals, ENVIRON HEALTH PERSP had the largest node (frequency = 1646), followed by ATMOS ENVIRON (frequency = 11,985), and SCI TOTAL ENVIRON (frequency = 1497).

**Table 6:** The top 10 co-cited journal.

Rank	Frequency	Centrality	Year	Cited journal	IF
1	1646	0.02	2013	ENVIRON HEALTH PERSP	9.031
2	1638	0.02	2013	ATMOS ENVIRON	4.108
3	1497	0.01	2013	SCI TOTAL ENVIRON	8.2
4	1338	0	2013	ENVIRON SCI TECHNOL	10.52
5	1189	0.01	2013	ENVIRON RES	7.587
6	1177	0	2013	ENVIRON INT	10.176
7	1127	0.01	2013	ENVIRON POLLUT	7.561
8	848	0	2013	ATMOS CHEM PHYS	5.135
9	843	0.01	2013	LANCET	81.716
10	811	0	2013	J AIR WASTE MANAGE	2.043

**Figure 7.**
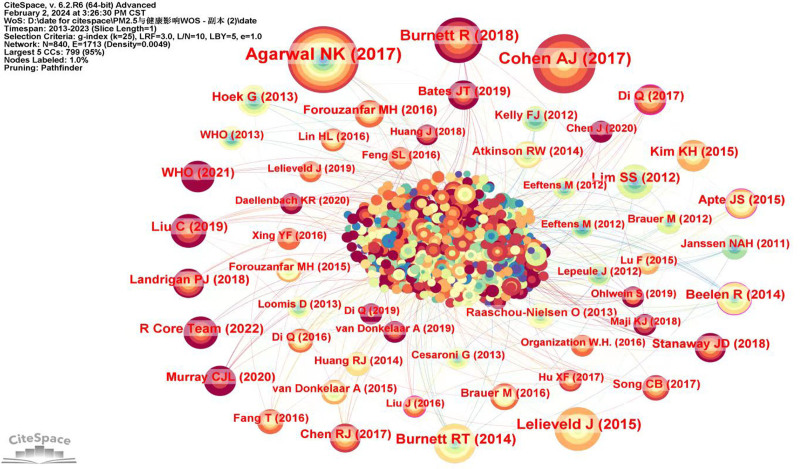
The network of co-cited references.

The co-citation analysis showed that 2 (or more) documents were cited by a third paper at the same time, and these 2 documents constitute a co-citation relationship, which greatly help researchers to identify the hotspots and trends in this field. Figure 7 shows the reference co-citation network map. Results showed that nodes N = 840, link E = 1713, and Density = 0.0049. Every node signifies a reference that has been co-cited; the more nodes there are, the more often they are mentioned, and the links show the relationship between co-citations across various articles.

The top 10 co-cited references ranked according to frequency are listed in Table 7. The first high-frequency co-cited reference highly cited primary literature was published by Agarwal et al in 2017.^[22]^ This article is an update of the American Heart Association’s first scientific statement on “Air Pollution and Cardiovascular Disease,” which provides a comprehensive review of new evidence linking PM exposure to cardiovascular disease and concludes that exposure to PM_2.5_ for hours to weeks can lead to cardiovascular disease-related deaths and nonfatal events. The second co-cited reference, authored by Cohen et al in 2017, highlighted ambient PM_2.5_ as the fifth most significant risk factor for mortality. Their study proved an increase in mortality is associated with PM_2.5_ exposure, as well as a rise in cases of COPD linked to ozone exposure.^[4]^ The third co-citation article was published by Burnett et al in 2018.^[23]^ This study aimed to improve the assessment of the risk of death from PM_2.5_ exposure. Finding that previous models may have underestimated the true disease burden, their study proposes the Global Exposure Mortality Model (GEMM), which predicts higher numbers of deaths based on data from cohort studies of outdoor air pollution. The fourth co-citation article published by Lelieveld et al in 2015^[24]^ is about the Global Burden of Disease Assessment study, which is based on an epidemiological cohort study that analyzed the relationship between air pollution and premature deaths. The study showed that about 3.3 million premature deaths per year globally are associated with outdoor air pollution, mainly due to PM such as PM_2.5_, and are concentrated in Asia. The fifth co-cited reference published by Burnett et al in 2014, fine particulate mass-based RR models covering the whole global range of environmental exposure to PM_2.5_ are developed by integrating RR data from various combustion sources that produce particulate emissions. The results of the paper improve the prediction abilities for the primary global causes of death from air pollution.^[25]^ The rest of the literature mainly focuses on the relationship between PM and mortality, analysis of the Global Disease Risk Assessment System (GDRAS), and the impact of atmospheric PM on human health.^[26–29]^

**Table 7:** Top 10 co-cited literature in frequency.

Rank	Frequency	Centrality	Year	Co-cited reference
1	210	0.09	2017	Agarwal NK, 2017, MED SCI, V21, P270, DOI 10.1161/CIR.0b013e3181dbece1
2	159	0.03	2017	Cohen AJ, 2017, LANCET, V389, P1907, DOI 10.1016/S0140-6736(17)30505-6
3	92	0.08	2018	Burnett R, 2018, P NATL ACAD SCI USA, V115, P9592, DOI 10.1073/pnas.1803222115
4	86	0.02	2015	Lelieveld J, 2015, NATURE, V525, P367, DOI 10.1038/nature15371
5	72	0.07	2014	Burnett RT, 2014, ENVIRON HEALTH PERSP, V122, P397, DOI 10.1289/ehp.1307049
6	54	0.01	2012	Lim SS, 2012, LANCET, V380, P2224, DOI 10.1016/S0140-6736(12)61766-8
7	53	0.07	2019	Liu C, 2019, NEW ENGL J MED, V381, P705, DOI 10.1056/NEJMoa1817364
8	49	0.04	2022	R Core Team, 2022, R LANG ENV STAT COMP, V0, P0
9	47	0.11	2014	Beelen R, 2014, LANCET, V383, P785, DOI 10.1016/S0140-6736(13)62158-3
10	45	0.09	2015	Kim KH, 2015, ENVIRON INT, V74, P136, DOI 10.1016/j.envint.2014.10.005

The top 10 co-cited references ranked according to betweenness centrality are listed in Table 8, The first co-cited reference studies how regional and global changes in ambient air quality could lower attributable mortality from PM_2.5_ by using cause-specific integrated exposure-response (IER) functions developed for the Global Burden of Disease 2010 in conjunction with high-resolution (10 km, world-coverage) concentration data.^[30]^ They show that effective programs to deliver clean air to the world’s most polluted regions could prevent hundreds of thousands of premature deaths each year. In the third co-cited reference, the IER model was used to assess the gridded premature mortality owing to PM_2.5_ exposure for adults in China who were 25 years of age or older.^[31]^ The fourth co-cited reference proves that among Medicare enrollees, low-income and self-identified minority groups have the most negative impacts from exposure to PM_2.5_ and ozone below existing national requirements.^[32]^ The eighth co-cited reference using meta-analyses found no correlation between nitrogen oxide concentration or traffic intensity on the closest street and lung cancer risk, but they did reveal a statistically significant relationship between risk for lung cancer and PM_10_.^[33]^ The ninth co-cited reference is an assessment of the global health burden associated with outdoor air pollution.^[34]^ The tenth co-cited reference summarizes a review of the effects of PM air pollution on human health, showing that PM is 1 of the most important components of air pollution and is associated with cardiovascular, cerebrovascular, and respiratory diseases.^[35]^

**Table 8:** Top 10 co-cited literature for centrality.

Rank	Frequency	Centrality	Year	Co-cited reference
1	39	0.13	2015	Apte JS, 2015, ENVIRON SCI TECHNOL, V49, P8057, DOI 10.1021/acs.est.5b01236
2	47	0.11	2014	Beelen R, 2014, LANCET, V383, P785, DOI 10.1016/S0140-6736(13)62158-3
3	21	0.11	2016	Liu J, 2016, SCI TOTAL ENVIRON, V568, P1253, DOI 10.1016/j.scitotenv.2016.05.165
4	41	0.1	2017	Di Q, 2017, NEW ENGL J MED, V376, P2513, DOI 10.1056/NEJMoa1702747
5	210	0.09	2017	Agarwal NK, 2017, MED SCI, V21, P270, DOI 10.1161/CIR.0b013e3181dbece1
6	45	0.09	2015	Kim KH, 2015, ENVIRON INT, V74, P136, DOI 10.1016/j.envint.2014.10.005
7	92	0.08	2018	Burnett R, 2018, P NATL ACAD SCI USA, V115, P9592, DOI 10.1073/pnas.1803222115
8	29	0.08	2013	Raaschou-Nielsen O, 2013, LANCET ONCOL, V14, P813, DOI 10.1016/S1470-2045(13)70279-1
9	26	0.08	2012	Brauer M, 2012, ENVIRON SCI TECHNOL, V46, P652, DOI 10.1021/es2025752
10	14	0.08	2012	Anderson JO, 2012, J MED TOXICOL, V8, P166, DOI 10.1007/s13181-011-0203-1

### 3.5. Co-occurring keywords and cluster analysis

**Figure 8.**
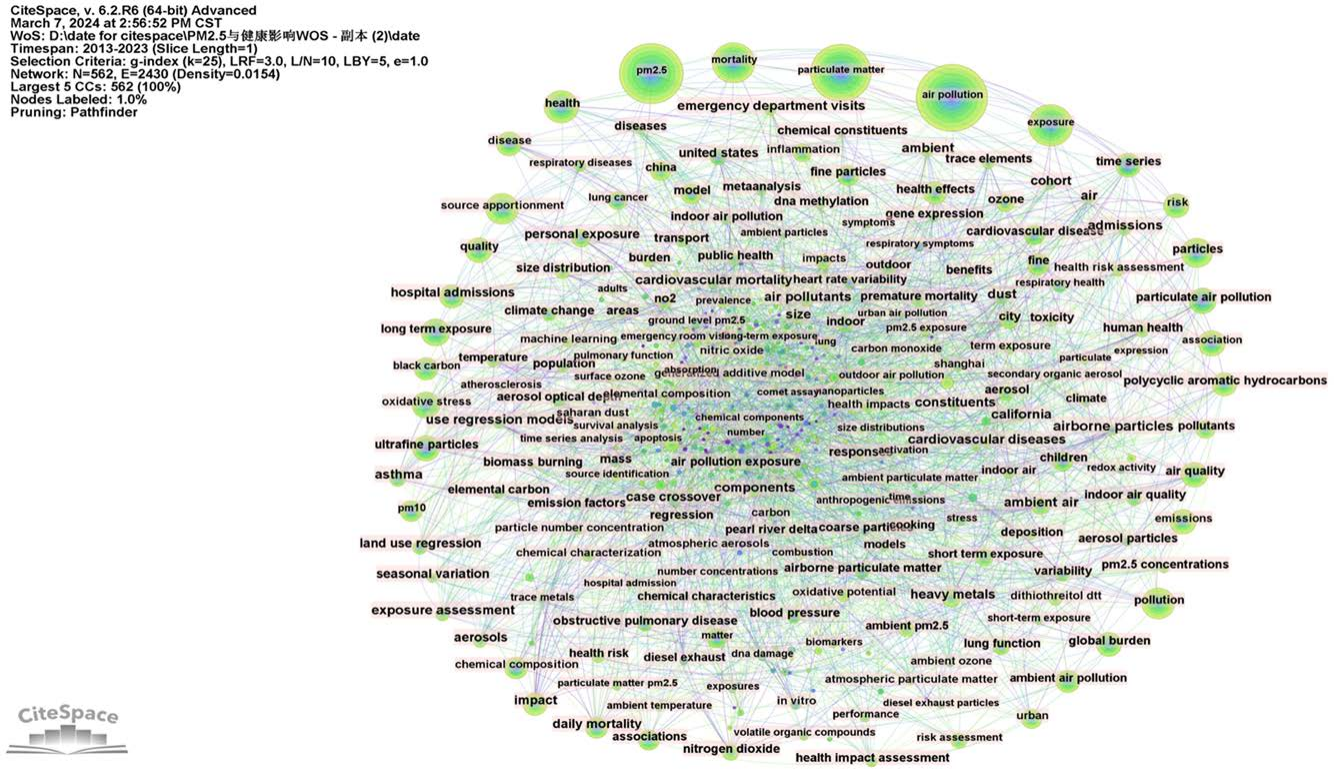
The network of co-occurring keywords.

In Figure 8, the co-citation network map for keywords is displayed. Results showed that nodes N = 562, link E = 2430, and Density = 0.0154. There were 562 keywords in all, and there was a lot of association between them. Research frontiers can be identified by analyzing the frequency and centrality of keywords.

Tables 9 and 10 display the top 10 keywords ranked based on frequency and centrality, respectively. The keywords that are prominent in this study include air pollution (frequency = 928), PM_2.5_ (frequency = 769), PM (frequency = 701), exposure (frequency = 416), mortality (frequency = 373), health (frequency = 246), source apportionment (frequency = 238), particles (frequency = 221), pollution (frequency = 220), and long-term exposure (frequency = 191). Keywords with high centrality in this study include air pollutants (Centrality = 0.08), ambient (Centrality = 0.06), airborne particles (Centrality = 0.06), size (Centrality = 0.05), California (Centrality = 0.05), model (Centrality = 0.04), asthma (Centrality = 0.04), and exposure assessment (Centrality = 0.04). This analysis sheds light on the significant role of air pollution, PM, and exposure in the context of ambient air quality. These findings suggest potential areas for future research exploration.

**Table 9:** Top 10 keywords in terms of frequency.

Rank	Frequency	Centrality	Year	Keywords
1	928	0	2013	Air pollution
2	769	0	2013	PM2.5
3	701	0	2013	Particulate matter
4	416	0	2013	Exposure
5	373	0.01	2013	Mortality
6	246	0.01	2013	Health
7	238	0.01	2013	Source apportionment
8	221	0.02	2013	Particles
9	220	0.02	2013	Pollution
10	191	0.02	2013	Long-term exposure

**Table 10:** Top 10 keywords for centrality.

Rank	Frequency	Centrality	Year	Keywords
1	30	0.08	2014	Air pollutants
2	42	0.06	2013	Ambient
3	20	0.06	2013	Airborne particles
4	73	0.05	2013	Ambient air
5	38	0.05	2013	Size
6	37	0.05	2013	Air
7	13	0.05	2014	California
8	92	0.04	2013	Model
9	74	0.04	2013	Asthma
10	56	0.04	2013	Exposure assessment

**Figure 9.**
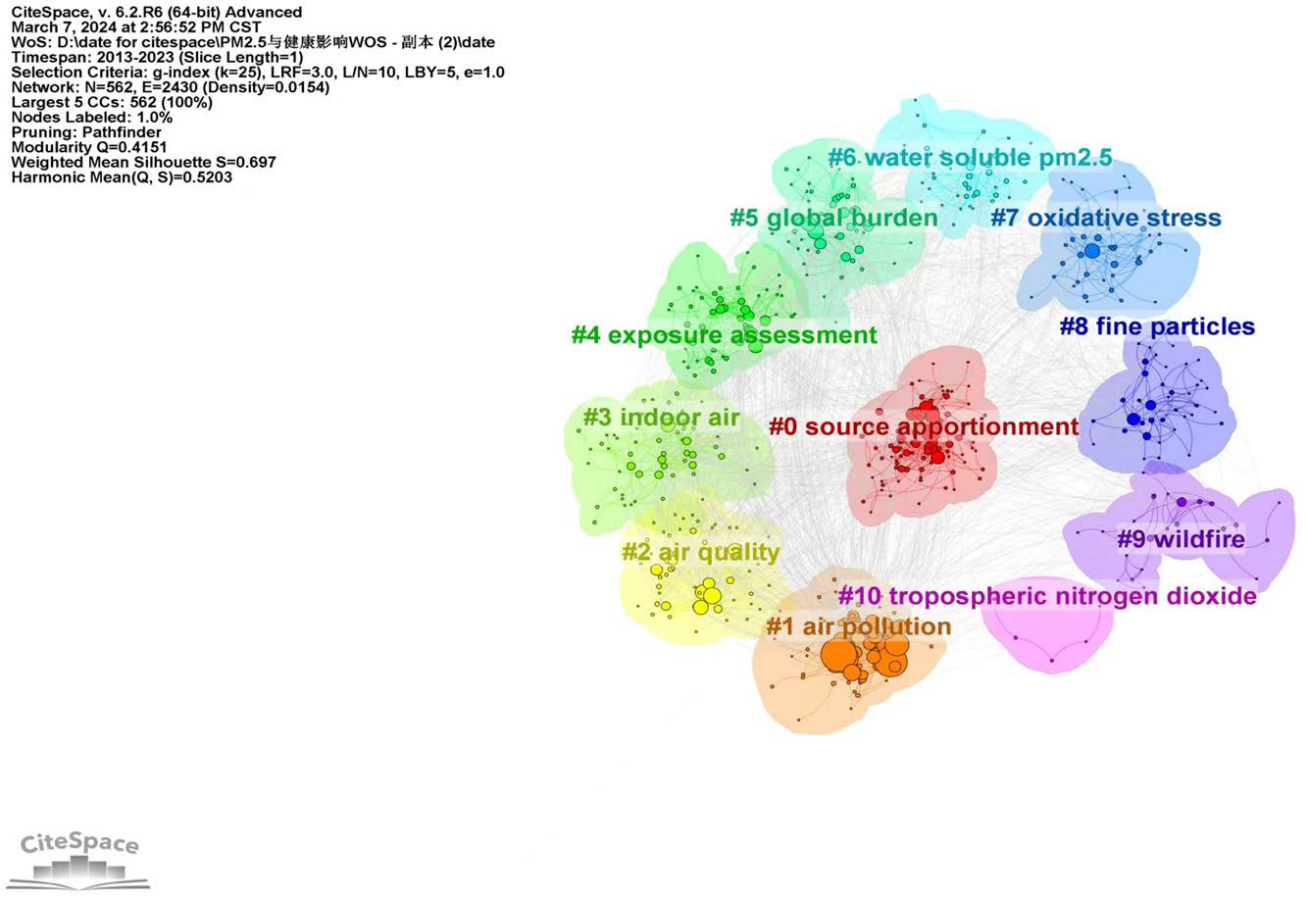
Keywords cluster analysis co-occurrence map.

The cluster analysis co-occurrence map of keywords is presented in Figure 9, revealing hotspots for research on PM_2.5_ and its health effects. The results of clustering indicate that there are 562 nodes and 2430 links, with a density of 0.0154, a modularity of 0.4151, and a weighted mean silhouette of 0.679. These values suggest that the clustering results are both reasonable and reliable. The 11 clustered tag terms are as follows: #0 source apportionment, #1 air pollution, #2 air quality, #3 indoor air, #4 exposure assessment, #5 global burden, #6 water-soluble PM_2.5_, #7 oxidative stress, #8 fine particles, #9 wildfire, and #10 tropospheric nitrogen dioxide. For further details on each cluster, refer to Table 11.

**Table 11:** Information of keyword clusters.

Cluster ID	Size	Silhouette	Mean (yr)	Top terms (log-likelihood ratio, *P*-level)
0	89	0.62	2017	Source apportionment (81.78, 1.0E−4); size distribution (51.92, 1.0E−4); chemical composition (37.34, 1.0E−4); trace metals (36.29, 1.0E−4); air pollution (33.42, 1.0E−4)
1	77	0.744	2015	Air pollution (93.26, 1.0E−-4); blood pressure (38.12, 1.0E−4); mortality (32.62, 1.0E−4); time series (31.72, 1.0E−4); lung function (26.69, 1.0E−4)
2	63	0.572	2017	Air quality (37.17, 1.0E−4); pollution (32, 1.0E−4); climate (26.74, 1.0E−4); model (23.02, 1.0E−4); air pollutants (19.95, 1.0E−4)
3	63	0.664	2017	Indoor air (22.96, 1.0E−4); human health (16.63, 1.0E−4); PM 2 (15.98, 1.0E−4); source apportionment (15.09, 0.001); environmental health (13.96, 0.001)
4	58	0.738	2016	Exposure assessment (53.02, 1.0E−4); land use regression (51.46, 1.0E−4); aerosol optical depth (43.11, 1.0E−4); machine learning (38.97, 1.0E−4); nitrogen dioxide (27.37, 1.0E−4)
5	57	0.645	2017	Global burden (41.41, 1.0E−4); lung cancer (39.29, 1.0E−4); long−term exposure (33.77, 1.0E−4); health impact assessment (33.77, 1.0E−4); public health (31.05, 1.0E−4)
6	44	0.753	2018	Water-soluble PM2.5 (47.21, 1.0E−4); humic like substances (41.29, 1.0E−4); airborne particulate matter (37.42, 1.0E−4); dithiothreitol DTT (35.38, 1.0E−4); secondary organic aerosol (35.38, 1.0E−4)
7	43	0.753	2016	Oxidative stress (43.45, 1.0E−4); cytotoxicity (41.65, 1.0E−4); inflammation (26.97, 1.0E−4); apoptosis (20.14, 1.0E−4); mortality (14.19, 0.001)
8	39	0.788	2015	Fine particles (36.48, 1.0E−4); coarse particles (18.6, 1.0E−4); air quality index (17.38, 1.0E−4); oxidative potential (15.73, 1.0E−4); ambient air (14.68, 0.001)
9	24	0.855	2018	Wildfire (15.33, 1.0E−4); climate change (11.32, 0.001); smoke (10.25, 0.005); ambient air quality (9.91, 0.005); (25) (9.91, 0.005)
10	5	1	2020	Tropospheric nitrogen dioxide (11.66, 0.001); aircraft operational data (11.66, 0.001); aerosol formation (11.66, 0.001); APU (11.66, 0.001); satellite AOD (11.66, 0.001)

### 3.6. Keywords with citation bursts

**Figure 10.**
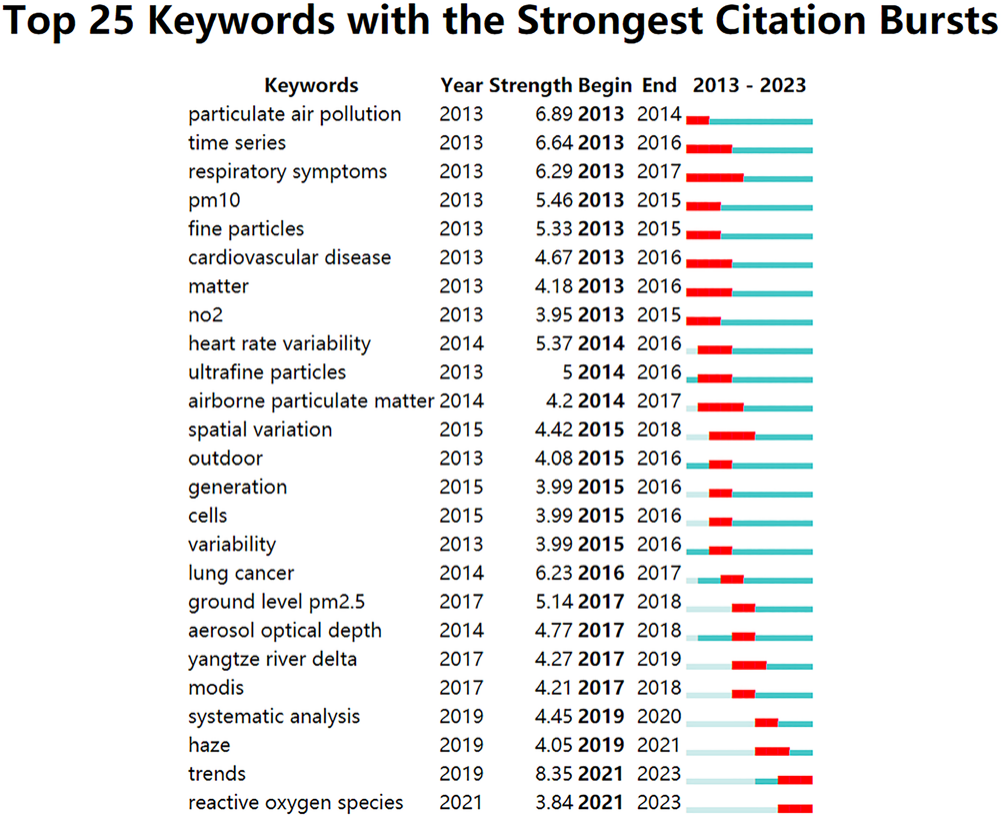
Top 25 keywords with the strongest citation bursts.

Figure 10 displays the top 25 keywords with the strongest citation burst for studies on PM_2.5_ and health effects from 2013 to 2023. The time axis is represented by the blue line, and the burst detection is shown by the red segment on the blue time axis, which shows the start year, end year, and burst duration.^[36]^ Citation bursts are a valuable tool for monitoring the rise or decline of a specific topic or term. Notably, trends (8.35) exhibited the strongest citation bursts, followed by particulate air pollution (6.89), time series (6.64), respiratory symptoms (6.29), lung cancer (6.23), PM_10_ (5.46), heart rate variability (5.37), fine particles (5.33), and ultrafine particles (5). Analyzing the starting time, it is evident that particulate air pollution, time series, respiratory symptoms, PM_10_, heart rate variability, and fine particles were among the early focal points of research. Currently, trends, lung cancer, aerosol optical depth, systematic analysis, haze, and ROS are leading the way in research on PM_2.5_ and its health effects.

## 4. Discussion

Chen developed the bibliometrics software CiteSpace, which is a tool for recognizing and presenting recent advancements and trends in the scientific literature. Its analysis data come from the Web of Science, and other database networks and can visualize the relationships in the literature in the form of scientific knowledge graphs, which not only aid in sorting out past research trajectories but also forecast prospects.^[37,38]^ As a result, we examine and visualize the nations, institutions, authors, journals, co-citations, keywords, etc using CiteSpace (version 6.2. *R*6).

The results of this study’s annual publication volume indicate a significant increase in PM_2.5_ and health effects research. The number of papers shows an upward trend, with 82 papers published in 2013, a relatively small number. The publication volume remained stable from 2013 to 2018, with a notable increase in relevant papers from 2019 to 2020. The peak number of papers was reached in 2021 with 292 publications, followed by a slight decline in 2022 and 2023. The rising trend of air pollution in various countries highlights the increasing importance of research on the relationship between PM_2.5_ and its health effects. China and the United States stand out as leading contributors to publications in this field, with a concentration of research institutions in these 2 countries. Notably, the Chinese Academy of Sciences (CAS) and Harvard University emerge as the most prolific institutions, indicating their central and authoritative positions within the collaborative network.

Hoek, Gerard is the author with the highest number of publications and his description is shown in coauthors and co-citation author network analysis above. The most co-cited author is Pope. Results of his studies have proved that acute exposure to PM_2.5_ has an impact on cardiovascular health. PM_2.5_ exposure can lead to elevated endothelial microparticle levels while affecting circulating levels of pro-angiogenic growth factors by decreasing levels of anti-angiogenic and pro-inflammatory cytokine levels and increasing levels of endothelial adhesion markers. In addition, PM_2.5_ may induce endothelial damage and systemic inflammation, which would impact cardiovascular health.^[39]^

The most frequently cited journal is ENVIRON HEALTH PERSP, an environmental health research journal sponsored by the National Institute of Environmental Health Sciences. The journal focuses on key research and cutting-edge advances in the fields of environmental sciences and public health, environmental health, and occupational health. This highlights the national influence of studies on PM_2.5_ and its health effects, emphasizing the importance of paying attention to this journal.

The results of keyword analysis reveal the key areas of focus in PM_2.5_ health effects research. The top ten keywords by frequency include air pollution, PM, PM_2.5_, exposure, mortality, health, source apportionment, particles, pollution, and long-term exposure. Additionally, cluster analysis identified 11 clusters, with #0 focusing on source apportionment and #6 on water-soluble PM_2.5_, indicating that researchers initially prioritize understanding the origins and composition of PM_2.5_. To effectively mitigate pollution and health risks associated with PM_2.5_, it is crucial to identify and address its sources. According to studies, the primary causes of PM_2.5_ include soil dust, secondary aerosol, coal combustion, biomass burning, and vehicle and industrial emissions.^[40,41]^ Secondary aerosols have been shown to predominate, highlighting the importance of controlling emissions of gaseous precursors in reducing PM_2.5_ concentrations.^[42]^ #1 Air pollution keywords include blood pressure, mortality, and lung function. Studies have investigated the relationship between atmospheric environment, specifically PM_2.5_, and blood pressure. It has been observed that exposure to atmospheric pollutants can trigger an inflammatory response in the body, affecting hemodynamics and potentially leading to hypertension. These research findings suggest that even minor changes in blood pressure and hypertension prevalence can have a significant impact on global mortality rates.^[43]^ #2 Air quality indicates that researchers have begun using models to analyze air pollutants. #3 Indoor air keywords include human health environmental health and source apportionment. Indoor air may contain a variety of harmful substances including, but not limited to, PM, volatile organic compounds (VOCs), formaldehyde, nitrogen oxides, and carbon dioxide.^[44]^ These pollutants originate from furniture, building materials, cleaning agents, tobacco smoke, gas, coal burning, etc, and long-term exposure may lead to a variety of health problems. Indicates that indoor air has an impact on human health and environmental health. #4 exposure assessment and #5 global burden indicate that long-term exposure to PM_2.5_ can lead to lung cancer and pose a global burden on public health. #7 oxidative stress and #8 fine particles keywords include oxidative stress; fine particles; and coarse particles highlight the attention given to the health hazards of small-sized PM. #9 wildfire and #10 tropospheric nitrogen dioxide PM_2.5_, wildfires, studies have shown that large amounts of nitrogen dioxide released by wildfires is 1 of the precursors of PM_2.5_, which undergoes a chemical reaction in the atmosphere to form fine PM. These PM_2.5_ PM deposits in the human respiratory tract and trigger an inflammatory response, which in turn increases the risk of cardiovascular and respiratory diseases and other health problems. Therefore, effective reduction of wildfires and control of tropospheric nitrogen dioxide emissions is 1 of the most important measures to reduce PM_2.5_-related health effects.

Research on PM_2.5_ and its health effects is focused on particulate air pollution, time series, respiratory symptoms, lung cancer, the Yangtze River Delta, and ROS. With the emergence of the term Yangtze River Delta in 2017 to 2019, the Yangtze River Delta region is 1 of the more economically developed regions in China. Still, it is also an important industrial and transportation hub with high PM_2.5_ particulate emissions. As a result, residents exposed to high levels of PM_2.5_ pollution for long periods may face a higher risk of respiratory diseases and lung cancers. Therefore, it is important to strengthen air quality management in the Yangtze River Delta region to reduce the emission of PM_2.5_ PM and protect the health of the residents. Haze also became a prominent term during the same period. Haze is produced by tiny particles of smoke, fog, and dust in the air.^[45]^ Haze is mainly composed of PM_10_ and PM_2.5_. Haze not only reduces visibility and increases the frequency of traffic accidents, but also leads to a reduction in air quality and induces respiratory and cardiovascular diseases. Researchers are actively studying the haze issue. From 2021 to 2023, the term reactive oxygen species (ROS) emerged.^[46–48]^ ROS, is a class of highly reactive oxidizing molecules or free radicals produced in living organisms, including superoxide anion (O_2_^−^), hydrogen peroxide (H_2_O_2_), and hydroxyl radical (OH^−^).^[49]^ There is a close relationship between ROS and disease. Excessive ROS can lead to cellular oxidative stress, which in turn damages cellular structure and function and triggers the onset and progression of a variety of diseases. Diseases commonly associated with ROS include cardiovascular diseases, neurodegenerative diseases, inflammatory diseases, tumors, and metabolic diseases.^[50]^ ROS pharmacotherapy refers to the use of drugs to intervene to regulate or control the process of generation and removal of ROS in the body, thereby treating ROS-related diseases. Specifically targeted drugs are designed and developed to target the mechanism of action of ROS in specific diseases or pathological processes. For example, for diseases such as neurodegenerative diseases or tumors, ROS-targeted drugs with specificity can be designed.^[51]^ Our previous study also proved the generation of ROS in hepatocytes is closely associated with its toxicity to liver cells while using oceanic acid-loaded nanoparticles can protect the liver by inhibiting ROS production.^[52]^ These drugs treat diseases by targeting the pathological process of the specific disease and the mechanism of action of ROS, modulating the generation and removal of ROS to reduce the harmful effects of ROS on cells. The development of such drug therapeutic strategies offers new possibilities for disease treatment and may become 1 of the most important means of treating various ROS-related diseases in the future. As a result, research is focused on understanding the mechanism of ROS action in the human body.

In this paper, a scientometric analysis was conducted using CiteSpace to enhance our systematic comprehension of the health impacts of PM_2.5_. While this approach has provided valuable insights, it is important to acknowledge its limitations. The data selection process relied on the WOS core collection, which only includes SCIE-Expanded and English-language papers, leading to a potential lack of comprehensive data. Additionally, the presence of various synonyms may result in overlapping terms when analyzing co-occurrence and clustering in the literature. Furthermore, this study focused primarily on articles, neglecting other sources of scientific knowledge dissemination such as books, working papers, and government reports. This narrow focus may have resulted in the exclusion of significant recent studies. To advance our understanding in this research area, future studies could consider utilizing a wider range of databases and analytical techniques.

## 5. Conclusions

This study examines the health impacts of PM_2.5_ using CiteSpace software to visualize and evaluate various years, nations, organizations, writers, journals, literature, and keywords in WOS 2013 to 2023. The visualization analysis reveals the following insights: Research on the health effects of PM_2.5_ has shown a rapid increase since 2018, peaking in 2021; Current focus areas include source analysis of PM_2.5_, health effects of nano/ultrafine particles, and their toxicological impacts. Studies have extensively explored the effects of PM_2.5_ on the respiratory and cardiovascular systems, as well as the prevention and treatment of related diseases. Additionally, there is a growing body of research on the mechanism of ROS in the human body.

## Acknowledgments

Prof Chao-Mei Chen, who created CiteSpace, for which there is no charge, is greatly appreciated by the authors.

## Author contributions

**Conceptualization:** Feifei Huang, Ming Li.

**Data curation:** Feifei Huang, Yao Lu, Ruiwei Liao.

**Formal analysis:** Lin Zhou, Yan Li.

**Methodology:** Feifei Huang, Ming Li.

**Software:** Lin Zhou, Yan Li.

**Visualization:** Feifei Huang, Lin Zhou, Yao Lu, Ruiwei Liao.

**Writing – original draft:** Feifei Huang, Ming Li.

**Writing – review & editing:** Lin Zhou, Yan Li, Ming Li.
